# Intermediate to Long-Term Follow-up of Distal Femoral Replacements in the Treatment of Neoplastic Disease About the Knee

**DOI:** 10.1016/j.artd.2021.03.014

**Published:** 2021-04-27

**Authors:** Evelyn P. Murphy, Sarah Conway, Christopher Fenelon, Peter H. Dawson, Gary C. O’Toole, Alan P. Molloy

**Affiliations:** a National Orthopaedic Hospital Cappagh, Dublin 11, Ireland; b St. Vincent’s University Hospital, Dublin 4, Ireland; c University of Limerick, Limerick, Ireland

**Keywords:** Distal femoral replacement, Megaprosthesis, Endoprosthesis, Tumor, Osteosarcoma

## Abstract

**Background:**

Limb salvage procedures have become more prevalent in orthopedic oncology. Endoprostheses have been used successfully to reconstruct large skeletal deficits. The aim was to review intermediate to long-term follow-up of distal femoral replacements in the setting of neoplastic disease about the knee.

**Methods:**

This was a single-center retrospective cohort study from 1997 to 2018 in a national referral center for oncology. The secondary objectives were to describe morbidity and mortality in this cohort. We recorded the modes of failure using Henderson classification system, complications, revisions, and all further operations.

**Results:**

Seventy-two distal femoral replacements were performed. Osteosarcoma was the most common indication (55 patients). Other indications included chondrosarcoma (7 patients), giant cell tumor (5 patients), Ewing’s sarcoma (2 patients), metastatic spread (2 patients), and leiomyosarcoma (1 patient). One-year mortality was 1.38% with an overall mortality of 13.8%, at the end of the study period. The 1-year revision rate was 4.2%, 30.5% for 10 years, and 38.8% for more than 15 years. The overall implant survival rate was 63.8%. The most common reasons for failure included aseptic loosening (16.6%), infection (16.6%), and local recurrence (9.7%) with an amputation rate of 6.9% in the cohort.

**Conclusion:**

Neoplastic disease of the lower limb is associated with significant morbidity. Aseptic loosening (16.6%) and infection (16.6%) were the most common reasons for failure in this cohort.

## Introduction

Distal femoral replacements (DFRs) are used in a variety of settings including oncology reconstruction, bone loss, revision arthroplasty, and periprosthetic fractures. Limb salvage procedures are becoming increasingly common because of improvements in diagnosis, treatment, and survival of patients with bone tumors. Major advances have been made in the field of orthopedic oncology, transforming conditions which historically would have had a 5-year survival of 22% to 58% [1]. Best practice dictates that these major reconstructive procedures are performed in specialized tertiary referral centers. This is important in the neoplastic cohort who often must undergo adjuvant treatments in line with best practice guidelines. However, the postoperative goals of weight-bearing and immediate stability remain the same between the neoplastic and revision cohorts. Prosthetic replacement allows the patients to mobilize early and regain functionality early, in potentially life-limiting conditions.

There is mixed evidence surrounding the survival of cohorts in patients with bone tumors. This may be due to small sample sizes, varying lengths of follow-up, and heterogeneity in treatment approaches [2]. Previous historical studies have reported on earlier designs of DFR from 1970s to 2000 [2]. Currently there are few large cohort studies reporting outcomes for DFRs using a modern design. The complication rates and survival rates differ in the neoplastic cohort from the elective arthroplasty and nonneoplastic cohort as these patients experience different challenges and have different comorbidities [1].

The aim of our study is to provide intermediate to long-term follow-up on the outcomes of DFR in the treatment of neoplastic disease in a tertiary referral center. The secondary objectives are to provide information on the modes of failure, complications, revisions, and all further operations associated.

## Materials and methods

This study was a single-center observational cohort study carried out in the Irish national tertiary center for neoplastic tumor surgery. The records are prospectively maintained by the tumor clinical nurse specialist. The inclusion criteria consisted of patients undergoing tumor resection and skeletal deficit reconstruction using a DFR. Patients with metastatic tumors, giant cell tumors requiring reconstruction or primary bone tumors, were eligible to be included. Exclusion criteria consisted of patients undergoing DFR for revision elective arthroplasty, nonneoplastic reasons, or less than 2 years of follow-up. A total of 85 patients who had DFRs in the national tumor hospital were initially identified; however, 11 were for revision elective arthroplasty, with 2 patients being excluded for follow-up less than 2 years.

Data on epidemiological parameters, tissue diagnoses, implant choice, complications, reasons for revision, and all-cause morbidity and mortality were collected. Radiographical analysis was available using the National Integrated Medical Imaging System. Patients were referred from other institutions and discussed at a multidisciplinary forum and treated according to established oncology protocols. Adjuvant chemotherapy was given if deemed appropriate according to protocols. Standard antibiotic prophylaxis was administered according to national guidelines and continued in the 24-hour postoperative period. We used a standardized operative technique. The patients were positioned supine, without tourniquet, and the incision incorporated the biopsy tract. The surgical approach most commonly used was a medial parapatellar approach. The resection was conducted respecting the principles as outlined by Enneking et al. [3]. The goal was for patients to progress to guided full weight-bearing with physiotherapy over a 6-week period. The patients were followed up with physical examination and radiographical analysis on a yearly basis, if appropriate.

The implants used in the study were custom-made Stanmore prosthesis, Biomet OSS system, the Kotz system, or the Stryker GMRS system. Failure was defined as requiring revision surgery. Henderson et al. described modes of failure for endoprostheses, and the etiology of failure was delineated in this fashion [4]. Henderson classified failures according to mechanical such as soft tissue, aseptic loosening, and structural failure and nonmechanical such as infection or tumor progression [4].

Descriptive statistics were reported as median with an interquartile range (IQR) reported. Epidemiological factors, surgical factors, and outcomes were recorded. The cumulative incidence for time to all cause revision, all cause reoperation, and revision for aseptic loosening were recorded. A survival analysis curve was reported for time to death by underlying diagnosis. Statistical analysis was conducted using SPSS version 26 (IBM Corp., Armonk, NY). Ethical approval was granted by institutional board review by the Cappagh National Orthopedic Hospital Ethics Research Committee.

## Results

### Study characteristics

**Figure 1:**
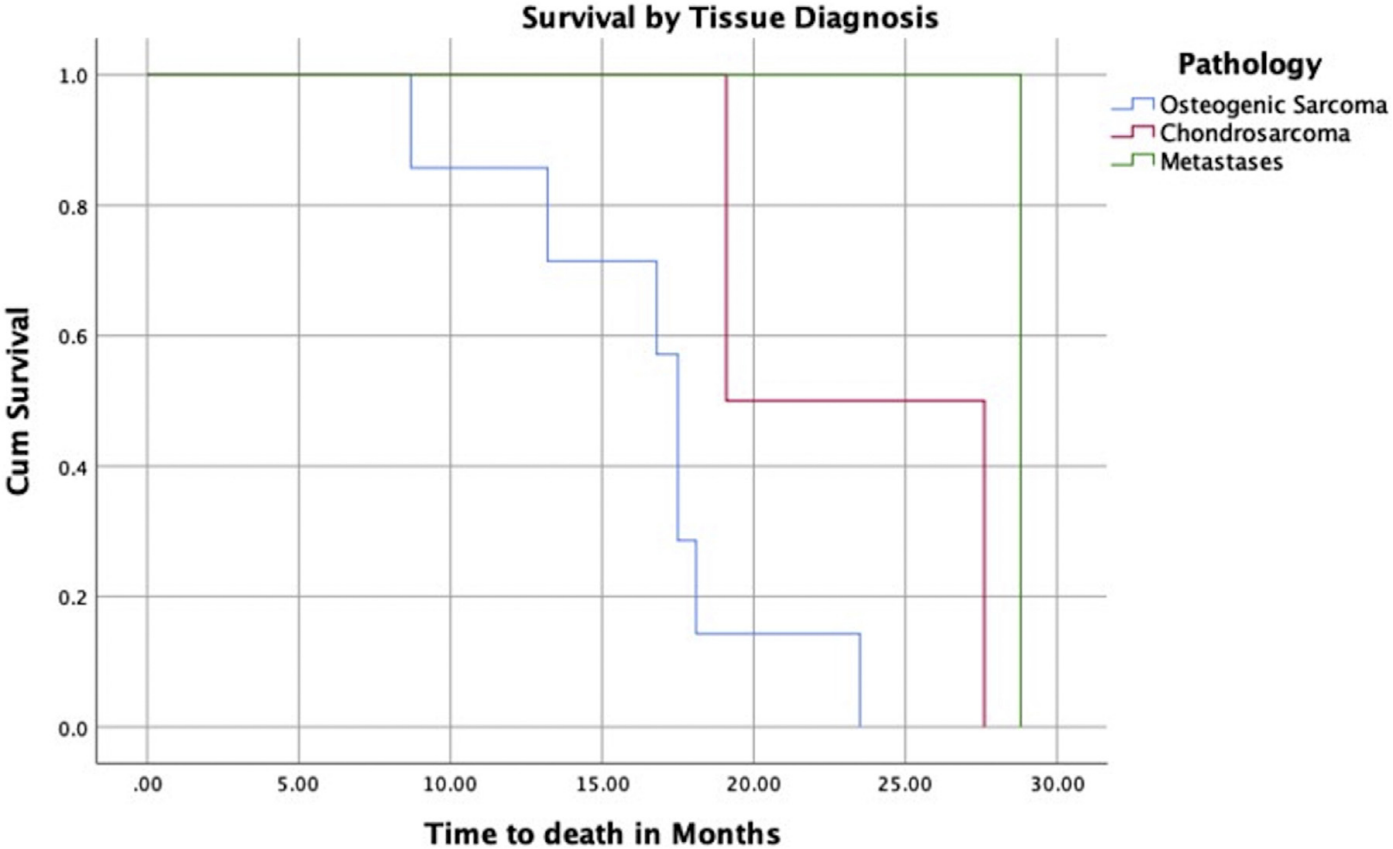
Survival analysis by diagnosis.

The study period ran from 1997 to 2018, and 72 patients underwent insertion of a DFRs, of which 56.9% (41 patients) were male (Table 1). The median follow-up was 11.3 years with an IQR of 9.25 years. Osteosarcoma was the most common indication for DFR followed by chondrosarcoma and giant cell tumor as seen in Table 2. The median age at the time of surgery was 31.8 years with an IQR of 13.2 years. At the end of the study, 62 (86.1%) patients were alive, with 10 deceased. Two patients were excluded with follow-up of less than 2 years, but all patients who died were included in the study. The median time to death was 3.1 years with an IQR of 1.1 years. One-year mortality was 1.38% with an overall mortality of 13.8%. The survival analysis curve demonstrates the time to death in Fig. 1.

**Table 1:** Tumor pathology diagnoses.

Pathology	N	%
Osteosarcoma	55	76.4%
Ewings sarcoma	2	2.8%
Chondrosarcoma	7	9.7%
Giant Cell tumor	5	6.9%
Leiomyosarcoma	1	1.4%
Metastasis	2	2.8%

**Table 2:** Epidemiological data.

Variables	N	%
Total patients	72	
Male	41	56.9%
Female	31	43.1%
Median age (years, IQ range)	31.8 (21-58.5)	
Alive at end of study	62	86.1%
Time to death (days, range)	1001.5 (250-750)

The overall complication rate including all-cause morbidity was 50% in this cohort (Table 3). The overall infection rate in the entire cohort was 16.6% (N = 12); 11 of these infections were deep infections (15.3%). The superficial infection occurred within 2 weeks, while the deep infections ranged from 3 months to 4 years. The amputation rate was 6.9% (N = 5) patients. Three of the amputations were for infection, and 2 amputations occurred for local recurrence.

**Table 3:** Complications and further surgeries.

Complications	N	%
Total number of complications	36	50%
Total number of implant revisions	28	38.8%
Revision for recurrence	7	9.7%
Revision for aseptic loosening	12	16.6%
Infection	12	16.6%
Implant failure related to bearing fracture	4	5.5%
Amputation	5	6.9%
Manipulation under anesthesia for stiffness	4	5.5%
Thromboembolic event	1	1.4%
Extensor mechanism problem	4	5.5%

There were 51 custom-made Stanmore prostheses (Stanmore Implants Ltd, London, UK), 12 Stanmore GMRS prostheses (Stanmore Implants Ltd, London, UK), 7 Biomet OSS prostheses (Biomet Inc, Warsaw, IN), and 2 Kotz modular prostheses (KMFTR, Howmedica GmbH, Kiel, Germany).

### Implant survival

The overall reoperation rate for the DFRs at the end of the study period was 41.8%. The 1-year revision rate was 4.2%, 18.1% fot 5 years, 29.1% for 10 years, and 38.8% for more than 15 years. The overall implant survival rate at the end of the study was 63.8%. The aseptic loosening rate requiring revision was 16.6% (N = 12). There was a 5.5% incidence of bearing surface failure (N = 4) which required revision. This included bushing failure and antirotation pin fracture. There was a 5.5% incidence of extensor mechanism problems (N = 4), which included patella subluxation and patella tendon avulsion. There were treated with soft tissue releases and suture repair of patellar tendon and postoperative bracing, respectively. There was a 9.7% reoperation rate for local recurrence (N = 7). This included further marginal excision. There was one pulmonary embolism (1.4%). Four patients (5.5%) required manipulation under anesthesia for stiffness. Table 4 demonstrates the modes of failure as defined by the Henderson classification.

**Table 4:** Henderson modes of failure.

Mechanical		Nonmechanical	
Soft tissue	4	Infection	12
Aseptic loosening	12	Tumor progression	7
Structural failure	4		

### Trends over the treatment period

The treatment challenges changed over the time frame of the study as depicted in Table 5. In the first decade of the study, infections and aseptic loosening were a more common cause for revision surgery. In the second decade of the study, mechanism and implant-related problems were a more common reason for revision surgery. The implant choice changed over the course of the study, with the custom-made prostheses being more commonly used in the first decade of the study. This is in part due to the implant availability at the time of the study inception and the improved access to modern design prostheses over time.

**Table 5:** Breakdown by decade of Henderson classification.

Breakdown	2000-2010				2010-2019			
	Custom-made Stanmore	GMRS	Biomet OSS	Kotz	Custom-made Stanmore	GMRS	Biomet OSS	Kotz
Infection	2	5	1			4		
Tumor progression	1	3				3		
Soft tissue		1	2			1		
Aseptic loosening	3	4		1		4		
Structural failure	1	1	1			1		

## Discussion

A key concern with respect to endoprosthesis in the lower limb is implant survival. The factors which govern implant survival include infection, constraint, and patient factors. The implant survival in this study at 10 years is 70.9%, which is in keeping with published literature for tumor cases. The main reasons for failure in this study group included aseptic loosening (16.6%) followed by infection (16.6%). The earlier studies by Bradish et al. demonstrate that implant survival can vary from 80% in a series of 40 nononcology patients to 87% (N = 37) in another more recent series by Berend and Lombardi [5,6]. A larger study by Wyles et al. in a nononcology cohort identified 144 DFRs, of which 11 were primary procedures [7]. The survival for all cause revision in this series was 72.5%. This is in contrast to the survival rates in oncology, as investigated by a systematic review by Haijie et al. which demonstrated short-term survival rates of 78.3% at 5 years, with a 10-year survival of 70.1% [8].

Toepfer et al. described a single-center cohort treated with DFR for oncology procedures, with a failure rate of 64% [9]. However, this study only had small numbers (36) in their cohort. These high rates of failure highlight potential areas for improvement. This study describes a change in challenges as the study progressed, with patients undergoing operations for stiffness or medial collateral ligamentous laxity in one case in the second decade.

Wound breakdown and infection are more common after oncology procedures than primary or revision arthroplasty. The quoted infection rates were investigated by Haijie et al. [8]. They highlighted an established rate of 8.5% in the literature. This can have devastating consequences and lead to amputation. The review did not find a difference between rotating or fixed bearing replacements for infection rates. A focus for future improvements must be on reducing the incidence of infection in this vulnerable cohort. Concerns with respect to infection can include tissue breakdown, skin flap viability, and postoperative dehiscence. Repeated operations can increase the chances of wound breakdown in an already vulnerable cohort.

Other groups (Toepfer et al.) described infection rates of 17.3% in their single-center cohort for oncology [9]. The rate in this cohort is 16.6% which is somewhat lower than the quoted rates for oncology. Wyles et al. describe a 27% rate of infection in a cohort of 111 nononcology patients. The breakdown includes 13 debridement, antiobiotic, implant retentions, 7 amputations for infection, and 10 revisions for PJI [7]. There is wide variation reported in the literature.

Myers et al. described a 10.7% amputation rate in their cohort of 335 patients (2). The amputation rate in this cohort is 6.9%. Han et al. undertook a meta-analysis which demonstrated that the survival rates at 5 years were improved in patients undergoing limb salvage vs primary amputation [10]. They analyzed a total of 1330 patients and found that there was no difference in the rates of recurrence between groups. There was improved survival in those able to undergo limb salvage surgery.

The modes of failure for the implants are described by Henderson et al. formally [4]. This cohort had a rate equal to that in the literature. Infection and aseptic loosening are generally recognized to be the more common causes of failure (Myers et al. and Henderson et al.) [2,4]. Two main types of implants exist, rotating and fixed hinge. A large study by Myers did not show much difference in the early stages of implant survival; however, implant fracture was more common in the rotating hinge group. Sixteen percent of the group required bushel replacement in Myers cohort (which was undertaken from 1978 to 2000), whereas only 5 (6.9%) required a change of bearing surfaces in this cohort. The study period in this cohort was from 2000 to 2019, perhaps reflecting changes in techniques or advances in prostheses. At the inception of this study, custom-made prostheses were being commonly used; however, there was a shift toward using non–custom-made toward the latter half of the study. A study by Chaudry et al. reviewed 76 hinged knee implants over a 7-year period and found that early failure was generally due to infection [11]. This study group included hybrid cemented and uncemented components. Pala et al. found that the most common reason for failure in their cohort of oncology and nononcology patients (175 patients) was infection at a rate of 9.3% [12]. Bettin et al. 2016 also found that DFRs could be used in trauma of the knee in elderly patients [13]. This cohort of 18 patients however had low functional demand and still incurred an 11% implant complication rate and a deep infection rate of 5.5%. Hart et al. in 2017 also compared a cohort of open reduction internal fixation vs acute DFR in 38 patients, 10 of which underwent DFRs [14]. The DFR can allow elderly patients to ambulate with all achieving walking in this group; while 1 in 4 in the open reduction internal fixation group became wheelchair dependent. The infection rate for the trauma group was 20% or N = 2. DFRs are a versatile implant, but the complications can be serious.

The strengths of this study include the long follow-up available for the cohort, through the national treatment center for neoplastic tumors. The study describes the challenges associated with treating this cohort and provides up-to-date information on the newer prostheses being used. This allows informed dialog between the physician and the patient. The limitations are the retrospective nature of this cohort. The study does not correlate American Society of Anesthesiologists grading with outcomes. There are different prostheses used in the cohort, but due to the small numbers in each prosthesis group, it was not possible to perform a more detailed statistical analysis.

## Conclusions

The use of DFRs provides consistent results for a challenging problem. Patients can expect 84.7% implant survival at 5 years, 73.6% survival at 10, 70.1% survival at 15 years, and 63.8% at greater than 15 years. The 1-year mortality rate was 1.38%, with the 5-year mortality being 11.1%.

Infection and aseptic loosening continue to be the 2 major threats for implant revision. The study population is generally younger than those receiving a DFR for other reasons such as periprosthetic fracture. The constrained nature of the implant leads to higher rates of aseptic loosening in this particular cohort. Overall, the struggle for the future is to reduce infection rates, while continuing to provide innovations with respect to the bearing surfaces. This study group demonstrates satisfactory outcomes with respect to medium-term and long-term outcomes for DFRs.

## Conflicts of interest

The authors declare that they have no known competing financial interests or personal relationships that could have appeared to influence the work reported in this article.

## Appendix A Supplementary data

Conflict of Interest Statement for Molloy

Conflict of Interest Statement for Fenelon

Conflict of Interest Statement for Murphy

Conflict of Interest Statement for O’Toole

Conflict of Interest Statement for Dawson

Conflict of Interest Statement for Conway
